# Robert Koch—His Work and Methods

**Published:** 1887-06

**Authors:** Geo. W. Lewis

**Affiliations:** Buffalo, N. Y.


### ROBERT KOCH—HIS WORK AND METHODS.

BY GEO. W. LEWIS, JR., A. M., M. D., BUFFALO, N. Y.

Ten years ago Dr. Robert Koch was living in comparative obscurity in one of the small towns of Germany, engaged in a work the nature of which attracted the attention of only a few widely-separated scientists. It was at a time when little or no reliance was placed in the feebly supported germ-theory of disease, and the few outbursts of evidence which tended to establish its truth were so much at variance with each other and with popular prejudice that but little headway was made. In a modest way he gave utterance to the results of his experiments, but his opinions passed comparatively unheeded until he demonstrated the so-called tubercle bacillus before a poorly attended meeting of scientists which convened in the city of Berlin. This served to arouse public interest, both in the man and in the subject upon which he had thrown a new light. During the following year his views were confirmed by other prominent workers in the field of bacteriology, and the tubercle organism soon came to be regarded as a reliable factor in the diagnosis of this disease.

He had outgrown the little town that fostered his early discoveries, and now felt the need of greater facilities for research and intercourse with the scientific world. He therefore located in Berlin, and shortly after became identified with the Imperial German Board of Health. Asiatic cholera next attracted his attention, and he spent the three following years in different parts of India, where the disease is epidemic most of the time. Together with a corps of worthy assistants, he visited every locality that seemed to favor its spread, with a view of ascertaining, if possible, the origin of the infectious material. Although he found that cholera was rife most of the year in Ceylon, Madras and Bombay, he was able to trace its transmission to these places to the active traffic between them and certain parts of the Delta of the Ganges. Further research satisfied him that this latter tract was the home of the disease, and his reasons for thus closely confining its habitat were based on the general character of the region.

The upper part of the Delta is densely inhabited, while the lower part or base of the triangle, which lies at a considerably lower level, is unapproachable to man on account of the inundations and the pernicious fevers which invariably attack any one who passes its borders. In this uninhabited part he found a luxuriant vegetation and an abundant variety of animal life. The products here exposed to putrefaction were nowhere more noticeable than on the boundary line between the inhabited and uninhabited parts, where the refuse from an extremely thickly populated country is floated down and mixes with the brackish water below, which flowing backwards and forwards is already saturated with putrefied matter. The entire stretch of country known as Lower Bengal is only slightly raised above the sea-level, and during the rainy season almost the whole extent is submerged. For this reason the inhabitants are compelled to build their huts upon raised ground. This is effected by taking the earth near where the hut is built, in order to raise the ground on which the house stands. The result of this displacement is to leave a large tank adjoining each hut, in which soiled water and putrefied matter from the household rapidly collect. Strange as it may seem, this very water is used for drinking and household purposes, and in turn receives much of the refuse which is necessarily thrown out.

Remaining in this locality several months, Dr. Koch was able to detect whatever slight fluctuations occurred, and was struck by the marked correspondence between these fluctuations and the prevalence of the disease in other parts of Europe. All European epidemics have been accompanied by a corresponding increase in the south of Bengal. During his stay, he performed over three hundred autopsies upon cholera corpses, and before leaving was successful in isolating from the discharges a micro-organism, which he called the Comma Bacillus of Asiatic Cholera. On returning to Berlin, he demonstrated the organism before the Imperial German Board of Health, and enlisted in his cause such men as Virchow, Gaffky, Grawitz, Pettinkoffer, Hueppe and other notable workers in the new field. They immediately set about confirming his observations, and with some slight variations arrived at his conclusions.

In view of the danger threatening the whole world, and the German nation in particular, the government now took up the subject, and deeming Kochs' experiments the most reliable, accorded to him the distinction of acquainting representative physicians from every city and town in Germany with the practical part of his theory. A large laboratory was fitted up, and delegations of ten physicians succeeded each other in a ten days' course, under his immediate supervision. This plan was followed until every town had sent at least one representative.

It is the arrangement and working of this laboratory system which the writer has been called upon to describe.

The entire second story of the Board of Health building is devoted to the laboratory purposes, and for convenience has been divided into two large working rooms, besides several smaller apartments for individual research. A single room, perhaps twelve feet square, with double walls between which is kept flowing a constant stream of corrosive sublimate solution (1 to 1000), is called the *Cholera-Zimmer*, and in it is placed the little jar of cholera dejecta, which is replenished from time to time from cholera-infected localities. This room is closely watched, and except at stated intervals no one is allowed admission. Another room is fitted up with the apparatus adapted to bacteriological study, such as steam and hot-air sterilizers, and the various materials used in the preparation of the cultivating media. The laboratory is heated by steam, and an even temperature maintained day and night.

The course which the writer was privileged to take, began on the 15th of December and closed on the evening of the 24th. The daily session lasted from six in the morning until dusk. On entering, we were assigned to private rooms, and each given a suit of stiffly-starched linen clothes, which we continued to wear during the working sessions of the entire course. Leaving all else behind, we proceeded to the so-called cooking room, where we found the various ingredients necessary for the preparation of the cultivating media already weighed out for us. These we combined and sterilized, according to a set of rules peculiar to Koch's system, and with which I presume most of my readers are now more or less conversant. It requires the better part of two days to make the nourishing substances, but these rather dry details over, the work is more interesting.

On the morning of the third day we made our first visit to the *Cholera-Zimmer.* In the centre of the room, on a plate of glass, stood the little tin box, about the size of a small pill box, containing the cholera discharges from which we were to make the inoculations. The glass plate was covered with filter-paper, and saturated with sublimate solution. After inoculating the tubes of food-gelatine, they were poured out in the liquid state upon sterilized glass plates, to allow the different species to colonize. By this means we were able to obtain a pure culture in about thirty-six hours. To impress the peculiar growth of this organism more strongly upon the mind, various other bacteria were cultivated at the same time, and the contrast between them made apparent by their manner of growth, rather than by their untrustworthy microscopic appearances.

In recalling the pleasant hours spent in this laboratory, it would be a gross oversight not to mention the midday lunch to which we were treated. From the time of entering in the morning until four or five o'clock in the afternoon, we were not allowed to leave the building. This, of course, necessitated some refreshment at midday, and in the good old German style it consisted of sandwiches and beer. The hours from ten to twelve each day were spent in the cholera room, and after passing through a thorough disinfection by means of extreme heat and cold, and a final washing in corrosive sublimate solution, we gathered about a long table, with Dr. Koch seated at the head. The conversation would naturally have to do with the morning's work, and to the writer the half hours thus spent proved by far the most interesting part of the course, taken up as they were with personal experiences, intermingled with rare bits of information pertaining to bacteriological study. The lunch over, the afternoons were given up to an examination of the cultures in their various stages of development, and the preparation of permanent microscopic slides.

The last three days were devoted to inoculation experiments upon rabbits, guinea-pigs, and white mice. As to the results of these experiments, I may say that they were successful only when the culture was introduced directly into the duodenum. When fed to the animals in appreciable quantities, no trustworthy symptoms were induced, and at the autopsy it was invariably found that the microbes had perished during their passage through the stomach. This bears out Koch's theory of the fatal action of the gastric juices upon this organism. It also closely follows our knowledge of the disease, for some impairment of the digestive process has always seemed the most important factor in cholera infection.

In personal appearance Dr. Koch is slightly above medium height, of rather stout habit, with dark complexion and prominent features. His eyes are deep set, the result, no doubt, of prolonged study over the microscope. This condition, of course, gives additional prominence to the cheek bones. His manner is one of retirement, and not at all calculated at first to inspire a feeling of ease on the part of those with whom he comes in contact. Later, however, this feeling gives place to a gradual and ever-increasing attachment. He is a man whose convictions are well defined and adhered to with almost obstinate tenacity. He possesses the two qualities essential to a successful mycologist—patience and perseverance. Combined with these is a rare trait, the ability to define one's position clearly, to which no doubt a large share of his present success is due.

